# Plasma homocysteine levels associated with a corrected QT interval

**DOI:** 10.1186/s12872-017-0617-z

**Published:** 2017-07-11

**Authors:** Zhao Li, Xiaofan Guo, Guozhe Sun, Liqiang Zheng, Yingxian Sun, Yamin Liu, Maria Roselle Abraham

**Affiliations:** 1 Department of Cardiology, the First Hospital of China Medical University, 155 Nanjing North Street, Heping District, Shenyang, 110001 People’s Republic of China; 2 Department of Clinical Epidemiology, Library, Shengjing Hospital of China Medical University, Shenyang, Liaoning People’s Republic of China; 3 Department of Cardiology, Johns Hopkins University, Baltimore, MD USA

**Keywords:** Homocysteine, Corrected QT interval, Metabolic syndrome, Cardiovascular disease

## Abstract

**Background:**

Little is known about the relationship between homocysteine (Hcy) levels and the QT interval. We examined the association of different Hcy levels with corrected QT (QTc) intervals in a general population.

**Methods:**

Plasma levels of Hcy were assessed in a population-based study of 7002 participants 35 years of age and older from 2012 to 2013. Twelve-lead ECGs were performed on all participants and analyzed automatically.

**Results:**

The distribution of Hcy levels was determined for an entire population after the data were grouped into quartiles (Q1: <=11.1umol/L; Q2: 11.1–13.8umol/L; Q3: 13.8–18.2 umol/L; Q4 > 18.2 umol/L). The mean value of the QTc interval in each quartile was 433.2 ± 23.8 ms, 430.0 ± 24.6 ms, 429.2 ± 24.5 ms and 430.6 ± 25.7 ms. Multiple logistic regression analyses showed that, compared with the second quartile, and after fully adjusting for potential confounding factors, the odds for QTc > 440 ms in the first and fourth quartile increased (*P* < 0.05), (OR: 1.23, 95% CI: 1.05–1.43 for Q1; OR: 1.40, 95% CI: 1.19–1.65 for Q4).

**Conclusions:**

QTc interval was associated with the Hcy level in this general population.

**Electronic supplementary material:**

The online version of this article (doi:10.1186/s12872-017-0617-z) contains supplementary material, which is available to authorized users.

## Background

Homocysteine (Hcy) is a non-protein amino acid that contains sulfur and is derived from the essential amino acid methionine [1, 2]. Previous studies reported that Hcy was associated with cardiovascular diseases such as aortic atherosclerosis, hypertension, coronary artery disease and acute myocardial infarction [3–9].

The QT interval is the electrocardiographic manifestation of ventricular depolarization and repolarization. A prolonged QT interval is closely related to ventricular arrhythmias, which can cause sudden cardiac death (SCD) [10]. In mice, the QT interval was found to be affected by Hcy levels [11]. Recent studies determined that Hcy was also related to arrhythmias and SCD, which indicated the possibility that Hcy was associated with cardiac electrophysiology [7, 8, 12–15]. However, few studies focused on the relationship between Hcy and the QTc interval in the general population. Detection of the QTc interval can help predict the risk of cardiovascular disease (CVD) events [16–19]. Therefore, in this study, we evaluated plasma Hcy levels and the QTc interval in a general population to determine whether there is an association between Hcy levels and the QT interval.

## Methods

### Study population

The methods used were described in previous studies [20, 21–23]. From January 2012 to August 2013, a representative sample of individuals at least 35 years of age was selected to describe the prevalence, incidence and natural history of cardiovascular risk factors in rural areas of the Liaoning Province, located in Northeast China. This study followed a random cluster-sampling scheme in 2 counties (Zhangwu and Liaoyang County) and one town was randomly selected from each county. From each town,8–10 rural villages were randomly selected, for a total of 26 rural villages. Participants that were pregnant, had malignant tumors and or mental disorders were excluded from this study. All eligible permanent residents aged at least 35 years of age from each village (total 8365) were invited to participate in the study, and response rate was 85.3%. We used baseline data from each participant with a complete data set sampled from Zhangwu and the Liaoyang counties, and a final sample size of 7002 (3260 men and 3742 women, Additional file 1). The study was approved by the Ethics Committee of China Medical University (Shenyang, China). All procedures were performed in accordance with ethical standards. Written consent was obtained from all participants after they had been informed of the objectives, benefits, medical terms and the personal information confidentiality agreement. If the participants were illiterate, we obtained written informed consent from their proxies.

### Data collection and measurements

The data collection and methods of measurements used in this study are as described in previous studies [22]. Data on demographic characteristics, lifestyle risk factors, dietary habits, family income, history of heart disease and medication used two weeks prior to the study were obtained through an interview with a standardized questionnaire. A central steering committee with a subcommittee for quality control was established. Dietary patterns were assessed using each participant’s recall of foods eaten during the previous year. The questionnaire included inquiries regarding the average consumption of several food items per week. The reported consumption was approximately quantified in terms of grams per week (i.e., vegetable consumption: rarely = 3, <1000 g = 2, 1000–2000 g = 1, ≥2000 g = 0; meat consumption including red meat, fish, and poultry: rarely = 0, <250 g = 1, 250–500 g = 2, ≥500 g = 3). Each participant was assigned a special diet score ranging from 0 to 6 calculated by the addition of their vegetable and meat consumption scores. Higher diet score values indicated higher meat consumption, lower vegetable consumption and greater adherence to a Westernized diet, while lower values indicated adherence to a Chinese diet. Similar methods for calculating diet scores can be found in the ATTICA study [24].

Blood pressure was measured three times at 2-min intervals after at least 5 min of rest according to The American Heart Association protocol. Blood pressure measurements were obtained using a standardized, automatic, electronic sphygmomanometer (HEM-907; Omron), which had already been validated according to the British Hypertension Society protocol [25]. The participants were advised to avoid caffeinated beverages or exercise for at least 30 min before the measurement. During the measurement, the participants were seated with their arms supported at heart level. The mean of three blood pressure (BP) measurements was calculated and used in all analyses. Body mass index (BMI) was calculated as weight in kilograms divided by the square of the height in meters.

For all participants, fasting blood samples were collected in the morning after at least 12 h of fasting. Blood samples were obtained from the antecubital vein using BD Vacutainer tubes containing EDTA (Becton, Dickinson and Co., Franklin Lakes, NJ, USA). Serum was subsequently isolated from the whole blood, and all serum samples were frozen at −20 °C for testing at a central, certified laboratory. Fasting plasma glucose (FPG), total cholesterol (TC), low-density lipoprotein cholesterol (LDL-C), high-density lipoprotein cholesterol (HDL-C), triglyceride (TG), serum potassium and magnesium and other routine blood biochemical indexes were analyzed enzymatically on an auto-analyzer (Olympus AU640 Auto-Analyzer; Olympus Corp., Kobe, Japan).

Plasma Hcy levels were measured by an enzyme cycling method using a Hitachi 7020 Automatic Analyzer (Hitachi). All laboratory equipment was calibrated, and blind, duplicate samples were used.

Standard 12-lead ECGs were conducted with a MAC 5500 (GE Healthcare, Little Chalfont, Buckinghamshire, UK) as previously described [22], and analyzed automatically with the MUSE Cardiology Information System, Version 7.0.0 (GE Healthcare). ECG parameters, including the QT interval, were measured automatically. The QT interval is measured from the earliest detection of depolarization in any lead to the latest detection of repolarization in any lead. Since the QT interval is influenced by heart rate, it is necessary to adjust heart rate for QT correction [26]. In this study, the QTc interval was corrected for the heart rate using the Bazett formula, which is the most common method [27–29]. Population were classified in two groups: one group included participants whose QTc intervals exceeding 440 ms, the other group included participants whose QTc intervals equal or less than 440 ms [30–32].

### Statistical analysis

Descriptive statistics were calculated for all variables, including continuous variables (expressed as the mean values and standard deviations) and categorical variables (expressed as numbers and proportions). Differences among categories were evaluated using the Student’s *t*-test, ANOVA, non-parametric tests, or the χ2-test. Scheffe method was used to adjust for multiple hypothesis tests when conducting pairwise comparisons of the QTc responses by homocysteine quartile. Multivariable logistic regression analyses were used to identify independent associations between Hcy quartiles, other factors, and QTc > 440 ms in different models. Odds ratios (ORs) and corresponding 95% confidence intervals (CIs) were calculated. Linear regression was also used to test the association between Hcy level and QTc interval. All statistical analyses were performed using SPSS Version 17.0 software, and *P* values less than 0.05 were considered to be statistically significant.

## Results

The present study of 7002 participants consisted of 3260 males and 3742 females. The mean participant age was 54 years, and the participant age ranged from 35 to 93 years. The mean Hcy level of the population was 17.32 ± 12.34 umol/L. Among the included population, 4695 and 2307 demonstrated QTc ≤ 440 ms and QTc >440 ms The distribution of Hcy levels was determined for the entire population after the data were grouped into quartiles: Q1 contained Hcy levels ≤11.1 umol/L; Q2 contained Hcy levels >11.1 umol/L and ≤13.8 umol/L; Q3 contained Hcy levels >13.8 umol/L and ≤18.2 umol/L; and Q4 contained Hcy levels >18.2 umol/L. All variables are presented in the Table 1. Except for sleep duration, serum potassium, and medication used, all variables were significantly different across the four groups (all *p* < 0.05).

**Table 1:** Baseline characteristics of study population according to homocysteine level (*N* = 7002)

Variables	Homocysteine in quartiles (mmol/L)				*P*-value
	Q1 (≤11.1)	Q2(>11.1 and ≤13.8)	Q3 (>13.8 and ≤18.2)	Q4 (>18.2)	
Age (year)	49 ± 9	54 ± 10	56 ± 10	57 ± 12	<0.001
Male gender	300 (17.2)	710 (40.8)	1044 (58.9)	1206 (69.4)	<0.001
Race of Han	1625 (92.9)	1660 (95.3)	1669 (94.2)	1618 (93.0)	0.01
Smoking	389 (22.2)	589 (33.8)	751 (42.4)	908 (52.2)	<0.001
Drinking	217 (12.4)	365 (21.0)	498 (28.1)	525 (30.2)	<0.001
Sleep duration (h/d)	7.6 ± 1.6	7.3 ± 1.6	7.3 ± 1.7	7.5 ± 1.8	0.122
Diet score	2.2 ± 1.2	2.2 ± 1.2	2.2 ± 1.2	2.3 ± 1.1	<0.001
Education					<0.001
Primary school or below	759 (43.4)	855 (49.1)	921 (52.0)	892 (51.3)	
Middle school	830 (47.5)	723 (41.5)	677 (38.2)	703 (40.4)	
High school or above	160 (9.1)	164 (9.4)	174 (9.8)	144 (8.3)	
Physical activity					<0.001
Low	391 (22.4)	373 (21.4)	420 (23.7)	478 (27.5)	
Moderate	1242 (71.0)	1259 (72.3)	1222 (69.0)	1176 (67.6)	
High	116 (6.6)	110 (6.3)	130 (7.3)	85 (4.9)	
Family income (CNY/year)					<0.001
≤ 5000	222 (12.7)	284 (16.3)	342 (19.3)	395 (22.7)	
5000–20,000	993 (56.8)	978 (56.1)	1000 (56.4)	986 (56.7)	
> 20,000	534 (30.5)	480 (27.6)	430 (24.3)	358 (20.6)	
BMI(kg/m^2^)	25.1 ± 3.9	24.7 ± 3.7	24.7 ± 3.8	24.8 ± 3.7	0.014
WC (cm)	82.7 ± 9.5	83.4 ± 10.0	83.7 ± 9.7	84.4 ± 10.1	<0.001
SBP (mmHg)	138.9 ± 23.4	140.4 ± 24.1	144.9 ± 24.3	148.1 ± 26.1	<0.001
DBP(mmHg)	80.0 ± 11.0	81.0 ± 11.8	82.4 ± 11.9	83.7 ± 12.5	<0.001
FPG (mmol/L)	5.9 ± 1.9	5.9 ± 1.8	5.8 ± 1.4	5.8 ± 1.6	0.002
TC (mmol/L)	5.0 ± 1.1	5.1 ± 1.0	5.1 ± 1.0	5.1 ± 1.0	0.009
TG (mmol/L)	1.6 ± 2.1	1.6 ± 1.3	1.7 ± 1.4	1.7 ± 1.6	<0.001
LDL-C (mmol/L)	2.8 ± 0.8	2.8 ± 0.8	2.8 ± 0.7	2.9 ± 0.8	<0.001
HDL-C (mmol/L)	1.5 ± 0.4	1.4 ± 0.4	1.4 ± 0.4	1.4 ± 0.4	<0.001
Serum calcium (mmol/L)	2.385 ± 0.105	2.382 ± 0.102	2.38 4 ± 0.106	2.396 ± 0.105	<0.001
Serum potassium (mmol/L)	4.189 ± 0.340	4.185 ± 0.327	4.185 ± 0.329	4.18 1 ± 0.364	0.72
Serum magnesium (mmol/L)	0.866 ± 0.073	0.879 ± 0.069	0.883 ± 0.073	0.878 ± 0.070	<0.001
History of heart disease^a^	138 (7.9)	180 (10.3)	196 (11.1)	163 (9.4)	0.01
Medication used^b^	936 (53.5)	933 (53.6)	979 (55.2)	981 (56.4)	0.243

Data are expressed as the mean ± SD or as n (%)

*Abbreviations*: *CNY* China Yuan (1CNY = 0.157 USD), *BMI* body mass index, *WC* waist circumference, *SBP* systolic blood pressure, *DBP* diastolic blood pressure, *FPG* fasting plasma glucose, *TC* total cholesterol, *TG* triglyceride, *LDL-C* low-density lipoprotein cholesterol, *HDL-C* high-density lipoprotein cholesterol

^a^Including coronary heart disease, arrythmia and heart failure

^b^Indicating any self-reported medication used in the past two weeks.ps:P for category from chi-square; for continous from non-parametric test

Table 2 presents the baseline characteristics of the study population according to the QTc interval. Participants with QTc > 440 ms had a greater history of heart disease, lower physical activity and medication use, but lower rates of current smoking and drinking than those participants with QTc ≤ 440 ms (all *P* < 0.001). Participants with QTc > 440 ms had higher BMI, SBP, DBP, FPG, TC, TG and LDL-C levels than those participants with QTc ≤ 440 ms (all *P* < 0.001). No significant differences in serum calcium or magnesium were observed between the two groups. These results were consistent with the results obtained using the other two methods for QT correction.

**Table 2:** Baseline characteristics of study population according to QTc (*N* = 7002)

Variables	QTc ≤0.44 s (*n* = 4695)	QTc > 0.44 s (*n* = 2307)	*P*-value
Age (year)	53 ± 10	56 ± 11	<0.001
Male gender	2589 (55.1)	671 (29.1)	<0.001
Race of Han	4394 (93.6)	2178 (94.4)	0.179
Smoking	1905 (40.6)	732 (31.7)	<0.001
Drinking	1250 (26.6)	355 (15.4)	<0.001
Sleep duration (h/d)	7.5 ± 1.7	7.4 ± 1.7	0.029
Diet score	2.3 ± 1.2	2.1 ± 1.1	<0.001
Education			<0.001
Primary school or below	2162 (46.0)	1265 (54.8)	
Middle school	2082 (44.3)	851 (36.9)	
High school or above	451 (9.6)	191 (8.3)	
Physical activity			<0.001
Low	969 (20.6)	693 (30.0)	
Moderate	3423 (72.9)	1476 (64.0)	
High	303 (6.5)	138 (6.0)	
Family income (CNY/year)			<0.001
≤ 5000	778 (16.6)	465 (20.2)	
5000–20,000	2664 (56.7)	1293 (56.0)	
> 20,000	1253 (26.7)	549 (23.8)	
BMI(kg/m^2^)	24.6 ± 3.7	25.2 ± 3.8	<0.001
WC (cm)	83.1 ± 9.8	84.5 ± 9.8	<0.001
SBP (mmHg)	139.9 ± 23.3	149.6 ± 26.3	<0.001
DBP(mmHg)	80.6 ± 11.2	84.3 ± 12.8	<0.001
FPG (mmol/L)	5.7 ± 1.4	6.1 ± 2.1	<0.001
TC (mmol/L)	5.0 ± 1.0	5.2 ± 1.1	<0.001
TG (mmol/L)	1.6 ± 1.5	1.9 ± 1.8	<0.001
LDL-C (mmol/L)	2.8 ± 0.7	2.9 ± 0.8	<0.001
HDL-C (mmol/L)	1.4 ± 0.4	1.4 ± 0.4	0.361
Serum calcium (mmol/L)	2.4 ± 0.1	2.4 ± 0.1	0.461
Serum potassium (mmol/L)	4.2 ± 0.3	4.1 ± 0.3	<0.001
Serum magnesium (mmol/L)	0.9 ± 0.1	0.9 ± 0.1	0.057
History of heart disease^a^	383 (8.2)	294 (12.7)	<0.001
Medication used^b^	2390 (50.9)	1439 (62.4)	<0.001

Data are expressed as the mean ± SD or as *n* (%)

*Abbreviations*: *CNY* China Yuan (1CNY = 0.157 USD), *BMI* body mass index, *WC* waist circumference, *SBP* systolic blood pressure, *DBP* diastolic blood pressure, *FPG* fasting plasma glucose, *TC* total cholesterol, *TG* triglyceride, *LDL-C* low-density lipoprotein cholesterol, *HDL-C* high-density lipoprotein cholesterol

^a^Including coronary heart disease, arrythmia and heart failure

^b^Indicating any self-reported medication used in the past two weeks.ps:P for category from chi-square; for continous from non-parametric test

**Fig. 1:**
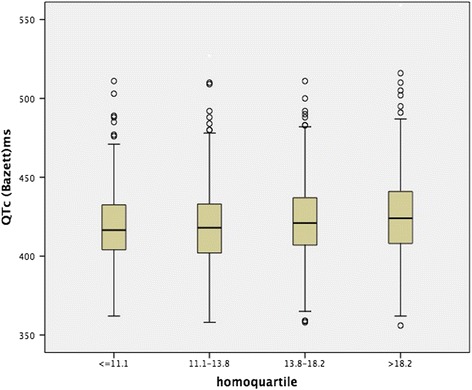
The QTc intervals of males according to the Hcy quartiles

**Fig. 2:**
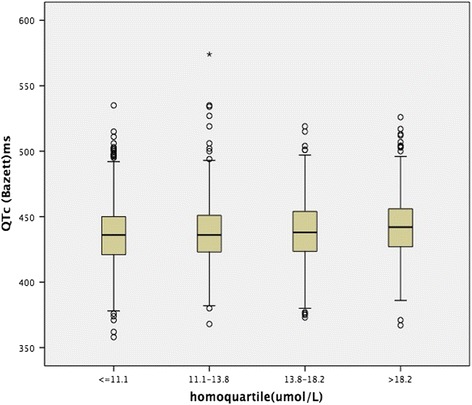
The QTc intervals of females according to the Hcy quartiles

**Fig. 3:**
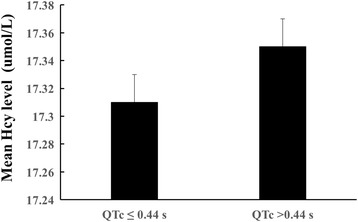
Mean Hcy levels in participants with QTc ≤ 440 ms and QTc > 440 ms

Figures 1 and 2 illustrates the QTc intervals according to the Hcy quartiles in boxplot. Using the Bazett formula. Regardless of the correction method, higher mean QTc values were observed in Q1 and Q4, compared to those in Q2 (*p* < 0.01). There were no significant differences in the mean QTc values between Q2 and Q3. And, as shown in Fig. 3, the mean Hcy levels among participants who had QTc > 440 ms were higher than those who had QTc ≤ 440 ms(*p* = 0.031).

Table 3 presents the relative odds of QT intervals. Using QTc intervals >440 ms as dependent variables and Hcy quartile as independent variables, multivariable logistic regression analyses were applied to assess the association between Hcy levels and the QTc interval. Model 1 was adjusted for age and gender, model 2 was adjusted for factors in model 1 and lifestyle variables including education level, family income, diet score, sleep duration, current smoking status, drinking status, and physical activity, model 3 was adjusted for factors in model 2 and CV risk factors included body mass index, waist circumference, systolic blood pressure, diastolic blood pressure, and total cholesterol, and model 4 was a multivariable model adjusted for factors in model 3 and other factors which was considered could empirically affect the Qtc directly including serum calcium, potassium and magnesium, history of heart disease, and any medication which . As shown in Table 3, in model 3, the risk of QTc intervals >440 ms in the first and fourth quartiles increased compared with those in the second quartile (*P* < 0.05). The odds ratio (OR) was 1.20 (95% CI 1.03–1.40) and 1.41 (95% CI 1.120–2.01). We also considered serum calcium, potassium and magnesium, history of heart disease, and any medication into the final, fully adjusted model (model 4) to rule out their impacts. The results were consistent. Compared with the second quartile, the risk of QTc intervals >440 ms in the first and fourth quartile increased (*P* < 0.05), and the OR was 1.23 (95% CI: 1.05–1.43) for Q1 and 1.40 (95% CI: 1.19–1.65) for Q4.

**Table 3:** Multivariable logistic regression analyses for association between homocysteine and long QTc interval

Homocysteine (quartiles)	Model 1			Model 2			Model 3			Model 4		
	OR	95% CI	*P*-value	OR	95% CI	*P*-value	OR	95% CI	*P*-value	OR	95% CI	*P*-value
Q1 (≤11.1)	1.205	1.038–1.399	0.014	1.196	1.030–1.390	0.019	1.203	1.031–1.404	0.019	1.226	1.049–1.432	0.010
Q2 (>11.1 and ≤13.8)		1.000 (reference)			1.000 (reference)			1.000 (reference)			1.000 (reference)	
Q3 (>13.8 and ≤18.2)	1.135	0.974–1.323	0.104	1.133	0.972–1.321	0.110	1.134	0.969–1.326	0.117	1.128	0.963–1.321	0.137
Q4 (>18.2)	1.504	1.287–1.758	<0.001	1.486	1.270–1.739	<0.001	1.408	1.197–1.656	<0.001	1.398	1.187–1.646	<0.001

Model 1: adjusted for age, sex and race

Model 2: adjusted for factors in model 1 and education level, family income, dietscore, sleep duration, current smoking, drinking status, and physical activity

Model 3: adjusted for factors in model 2 and and body mass index, waist circumference, systolic blood pressure, diastolic blood pressure, total cholesterol

triglyceride, low-density lipoprotein cholesterol, high-density lipoprotein cholesterol, fasting plasma glucose

Model 4: adjusted for factors in model 3 and serum calcium, potassium and magnesium, history of heart disease and any medication

*Abbreviations*: *QTc* corrected QT, *OR* odds ratio, *95% CI* 95% confidence interval

In the Additional file 2, we presented the result from linear regression analyses for association between homocysteine and QTc interval.

## Discussion

In the present study, we evaluated the relationship of different Hcy levels to the QTc interval in the general population. We found that the odds for QTc > 440 ms in the first and fourth quartile of the Hcy level increased compared with those in the second quartile.

Prior studies explored the relationship between Hcy levels and arrhythmias. Duyuler et al. found an independent relationship among the iron status, Tp-e interval and Tp-e/QT ratios of elite sport players that were also not correlated with serum homocysteine levels [33]. Previous researchers also found that the homocysteine levels determined prior to electrical cardioversion could predict recurrence of AF after successful restoration of sinus rhythms [34]. However, few studies focused on the QTc interval. Rosenberger et al. found a highly significant prolongation of the QTc interval in male C57/BL6J mice that received standard rodent chow and drinking water supplemented with 400 mg DL-homocysteine compared with the controls, which received standard rodent chow and water ad libitum [11]. Acampa et al. measured Hcy plasma levels, P wave dispersions (PWD), and the QTc interval in 32 patients who had orthotropic heart transplantation (OHT) and in 20 control subjects. Acampa found that, in OHT patients, plasma Hcy levels significantly correlated with PWD, whereas no correlation was found with the QTc interval [13]. Lee Yin Leng et al. concluded that in community populations, homocysteine was moderately elevated when QRS durations were >120 ms, but QTc intervals were not significantly associated with plasma homocysteine [26]. In our study, Linear regression and multiple logistic regression analyses results showed an association between QTc interval and Hcy levels. Similar to other studies in China [35], we obtained the elevated average level of Hcy in this general population. Some studies reported a high prevalence of HHCY in Chinese populations, and smoking status, alcohol consumption, education level, physical activity and MTHFR genotypes were significant determinants. These factors also may explain the high levels of Hcy in this study.

To the best of our knowledge, this study is the first to demonstrate a correlation between Hcy levels and the QTc interval in a general population. Our results confirmed that a link exists between Hcy levels and cardiac electrophysiology. However, the mechanism of Hcy-mediated cardiac arrhythmias is still unclear. According to laboratory studies, elevations in Hcy levels can inhibit cardiac K1 channels, which may delay ventricular repolarization [8, 36]. Homocysteine may decrease the Na + channel activity in in vitro cardiac preparations [9, 37–39]. Moreover, biochemical damage from high homocysteine levels on the atrial extracellular matrix involves the activation of matrix metalloproteinases-9 and extracellular signal regulated kinase and causes subsequent atrial fibrosis with slow and a heterogeneous atrial conduction, favoring the appearance of a vulnerable reentrant substrate [40–42]. However, these results do not explain the link between Hcy levels and the QTc interval. Therefore, future research is needed regarding this relationship. In addition, research is necessary to identify a mechanism that explains how both higher and lower levels of Hcy affect the QTc interval.

Our study had some limitations. First, we sampled adults only in the Liaoning Province, and could expect varying results if we had sampled adults in other Chinese provinces. Second, electrocardiograms for the participants were performed at one time, which may have affected our results. In addition, our results were based on a cross-sectional design, and thus no cause-and-effect relationships could be established. Longitudinal studies are required for further investigation of these findings. And, in this study we used 440mms as the cut off, we agree with that opinion that dichotomization is debatable and variability of inter observer cannot be completely avoided. However, the linear regression gave the similar result. In this paper we can only present an association between homocysteine levels and QTc but can’t conclude the correlation between homocysteine and prolonged QTc. Finally, beats with arrhythmias including AV-blocks were not deleted from QT measurements. Even though we used the heart rate with the correct QT interval, the effects of irregular heart rates could not be avoided.

## Conclusion

In this general population, Hcy levels are associated with the QTc interval. The result provides novel, population-based evidence of the relationship between Hcy levels and the QTc interval.

## Additional files

Additional file 1: Supplementary flow chart. Baseline data from each participant with a final sample size of 7002 (3260 men and 3742 women). (DOC 41 kb)

Additional file 2: Linear regression analyses for association between homocysteine and QTc interval. Linear regression analyses presented the result that homocysteine is associated with QTc interval. (DOCX 69 kb)

## Abbreviations

BMI: body mass index
CVD: cardiovascular disease
DBP: diastolic blood pressure
Hcy: Homocysteine
QTc: corrected QT
SBP: systolic blood pressure
SCD: sudden cardiac death.
