# Supplementary material for: Plasma homocysteine levels associated with a corrected QT interval
Source: BMC Cardiovasc Disord. 2017 Jul 11;17:182. doi: 10.1186/s12872-017-0617-z (PMC5504627; doi:10.1186/s12872-017-0617-z)
Supplement: Supplementary file 1 — Supplementary flow chart. Baseline data from each participant with a final sample size of 7002 (3260 men and 3742 women). (DOC 41 kb) [file 12872_2017_617_MOESM1_ESM.doc]

**Supplementary flow chart**

8365 individuals in two towns aged 35 years and older included between January 2012 to August 2013.

7135 participants joined in the study

7002 participants in final

analyses (3260 men and 3742 women)

133 with missing

laboratory values

Respond rate is 85.3%,1230residents didn’t joined in.
