# Supplementary material for: Plasma homocysteine levels associated with a corrected QT interval
Source: BMC Cardiovasc Disord. 2017 Jul 11;17:182. doi: 10.1186/s12872-017-0617-z (PMC5504627; doi:10.1186/s12872-017-0617-z)
Supplement: Supplementary file 2 — Linear regression analyses for association between homocysteine and QTc interval. Linear regression analyses presented the result that homocysteine is associated with QTc interval. (DOCX 69 kb) [file 12872_2017_617_MOESM2_ESM.docx]

| **Supplementary table**  **Linear regression analyses for association between homocysteine and QTc interval** | | | | | |
| --- | --- | --- | --- | --- | --- |
|  |  |  |  |  |  |
|  | **Standardized Coefficients** | **t** | **Sig.** | **95.0% Confidence Interval for B** | |
|  | **Beta** |  |  | **Lower Bound** | **Upper Bound** |
| **Constant** |  | 53.64 | 0.000 | 399.35 | 429.65 |
| **Male gender** | 0.34 | 23.66 | 0.000 | 15.54 | 18.35 |
| **Age (year)** | 0.15 | 11.94 | 0.000 | 0.29 | 0.40 |
| **Race of Han** | 0.01 | 0.88 | 0.377 | -1.22 | 3.21 |
| **smoking** | 0.02 | 1.48 | 0.138 | -0.29 | 2.13 |
| **drinking** | 0.02 | 1.71 | 0.087 | -0.19 | 2.89 |
| **totalsleep** | 0.02 | 1.59 | 0.112 | -0.06 | 0.57 |
| **dietscore** | 0.00 | -0.22 | 0.828 | -0.52 | 0.42 |
| **Physical activity** | -0.04 | -3.74 | 0.000 | -3.03 | -0.94 |
| **Family income (CNY/year)** | -0.02 | -1.43 | 0.152 | -1.48 | 0.23 |
| **FPG (mmol/L)** | 0.11 | 9.92 | 0.000 | 1.30 | 1.94 |
| **TC (mmol/L)** | -0.08 | -2.71 | 0.007 | -3.10 | -0.50 |
| **TG (mmol/L)** | 0.10 | 5.83 | 0.000 | 1.00 | 2.02 |
| **LDL-C (mmol/L)** | 0.05 | 3.57 | 0.000 | 1.46 | 5.00 |
| **HDL-C (mmol/L)** | 0.10 | 3.99 | 0.000 | 1.56 | 4.56 |
| **Serum calcium (mmol/L)** | -0.02 | -1.46 | 0.145 | -9.94 | 1.47 |
| **Serum phosphorus (mmol/L)** | -0.01 | -1.09 | 0.276 | -5.19 | 1.48 |
| **Serum magnesium (mmol/L)** | 0.03 | 2.36 | 0.019 | 1.56 | 17.06 |
| **Serum potassium (mmol/L)** | -0.14 | -12.24 | 0.000 | -11.81 | -8.55 |
| **Hcy (umol/L)** | 0.06 | 4.99 | 0.000 | 0.07 | 0.16 |
| **HeartDiseaseHistory** | 0.04 | 3.25 | 0.001 | 1.19 | 4.80 |
| **anymedicinehistory** | 0.05 | 4.51 | 0.000 | 1.42 | 3.59 |
